# Supplementary material for: Does Postoperative Oral and Intestinal Microbiota Correlate with the Weight-Loss Following Bariatric Surgery?—A Cohort Study
Source: J Clin Med. 2020 Nov 27;9(12):3863. doi: 10.3390/jcm9123863 (PMC7761068; doi:10.3390/jcm9123863)
Supplement: Supplementary file 1 [file jcm-09-03863-s001.pdf]

Supplementary Material

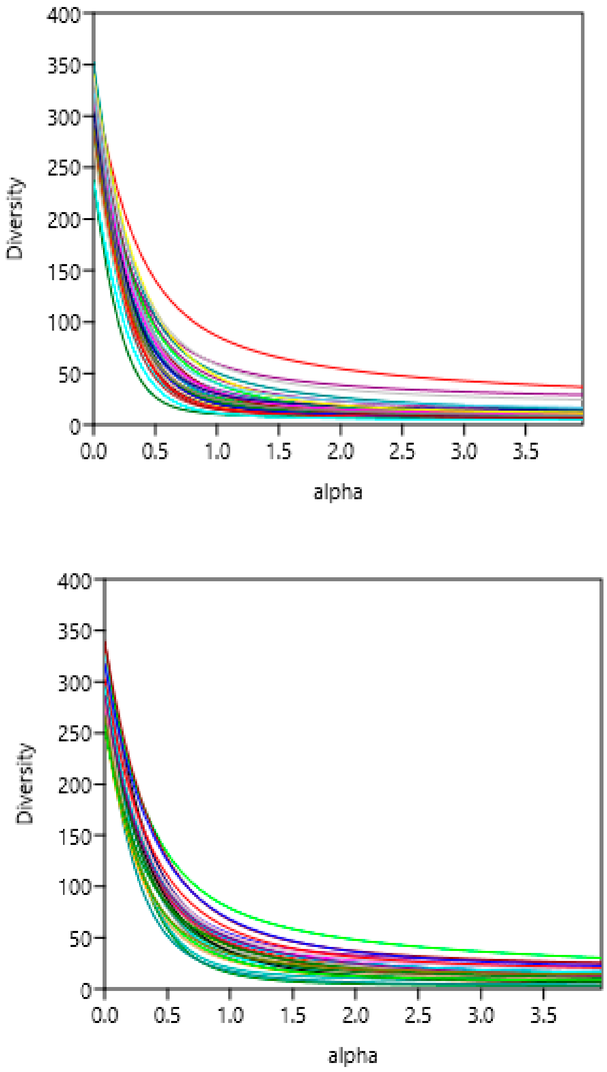

Figure S1. Alpha diversity for oral and intestinal microbiota.

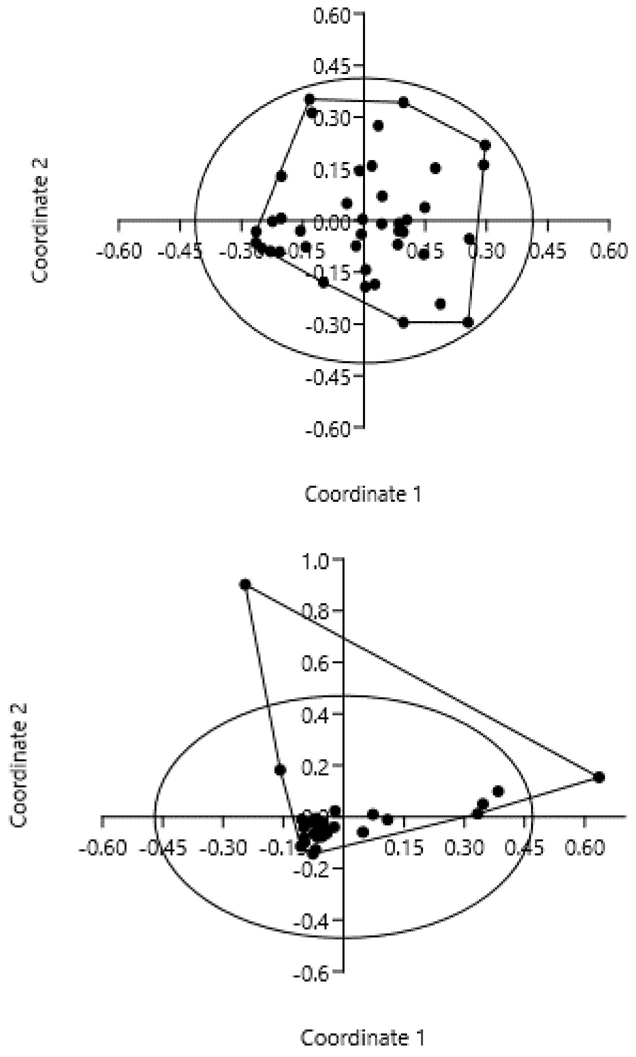

Figure S2. Beta diversity for oral and intestinal microbiota.

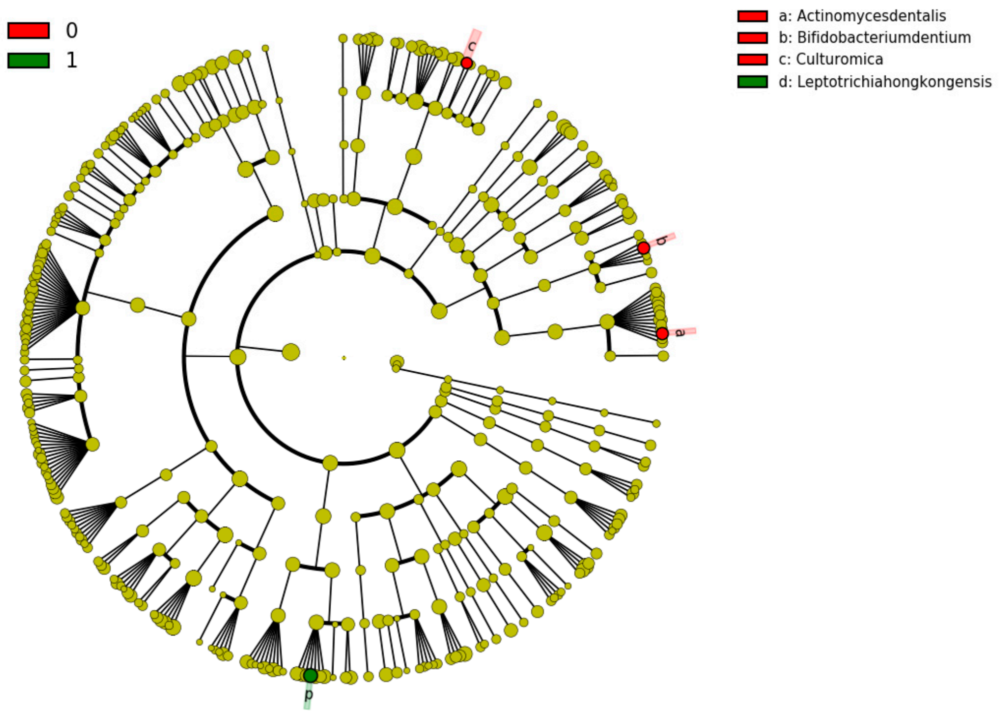
0
1
a: Actinomycesdentalis
b: Bifidobacteriumdentium
c: Culturomica
d: Leptotrichiahongkongensis

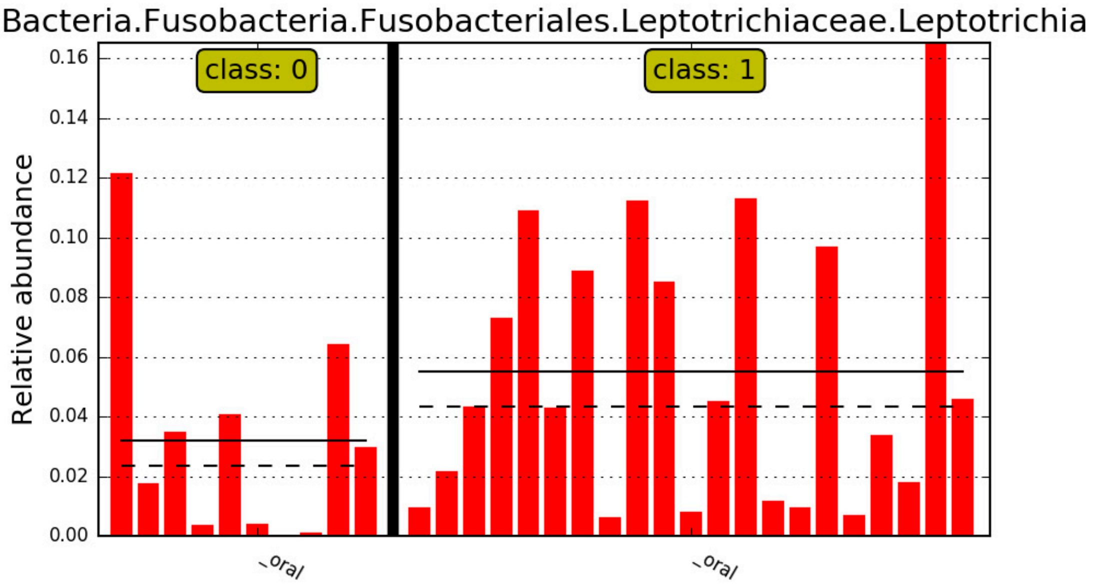
Bacteria.Fusobacteria.Fusobacteriales.Leptotrichiaceae.Leptotrichia
class: 0
class: 1
Relative abundance
0.00
0.02
0.04
0.06
0.08
0.10
0.12
0.14
0.16
_oral
_oral

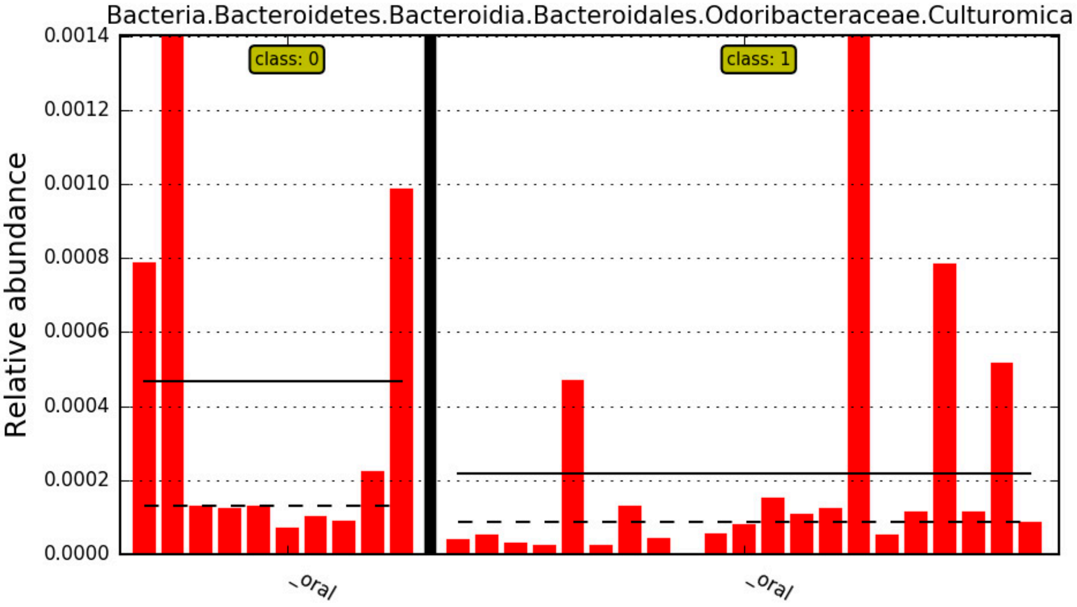

Figure S3. Differences in oral microbiota between Group 1 and Group 2 (1-Group 1; 0-Group 2).

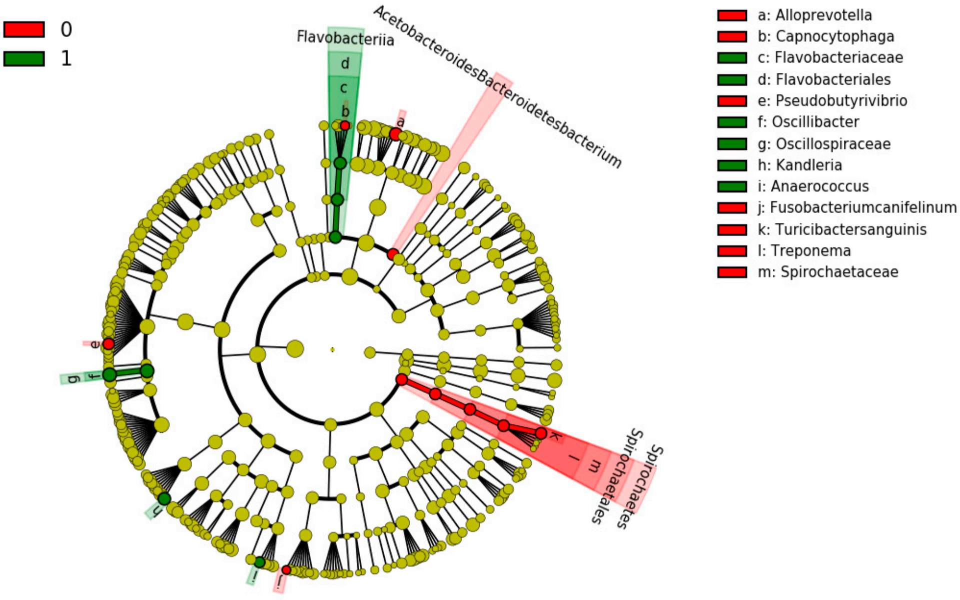

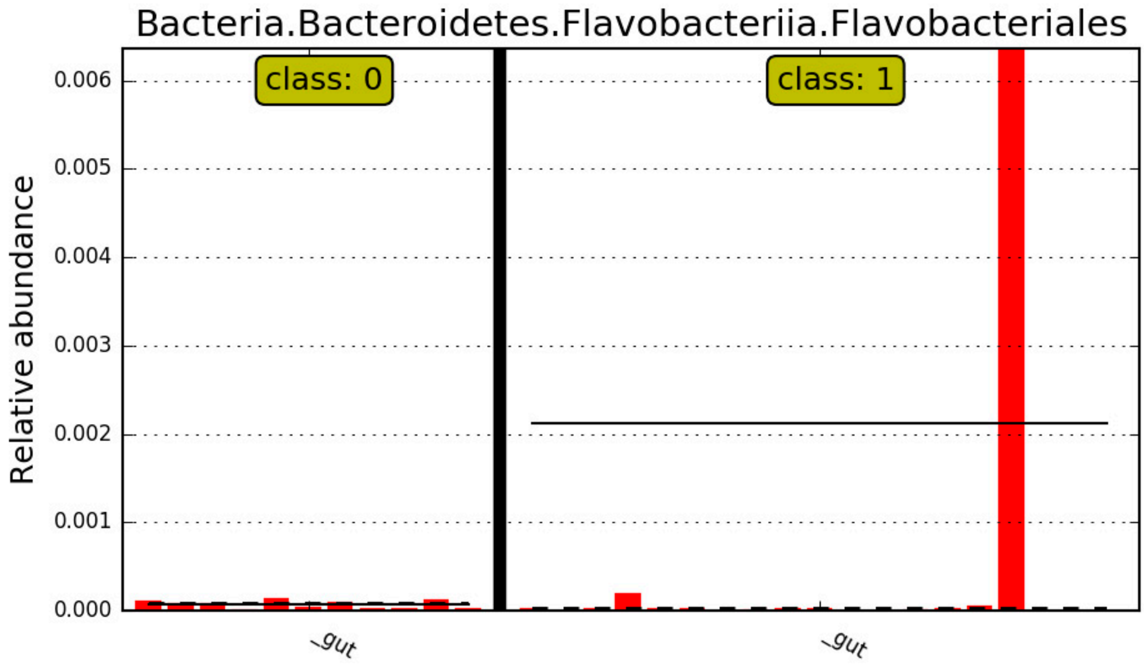
Bacteria.Bacteroidetes.Flavobacteriia.Flavobacteriales
Relative abundance
0.006
0.005
0.004
0.003
0.002
0.001
0.000
class: 0
class: 1
_gut
_gut

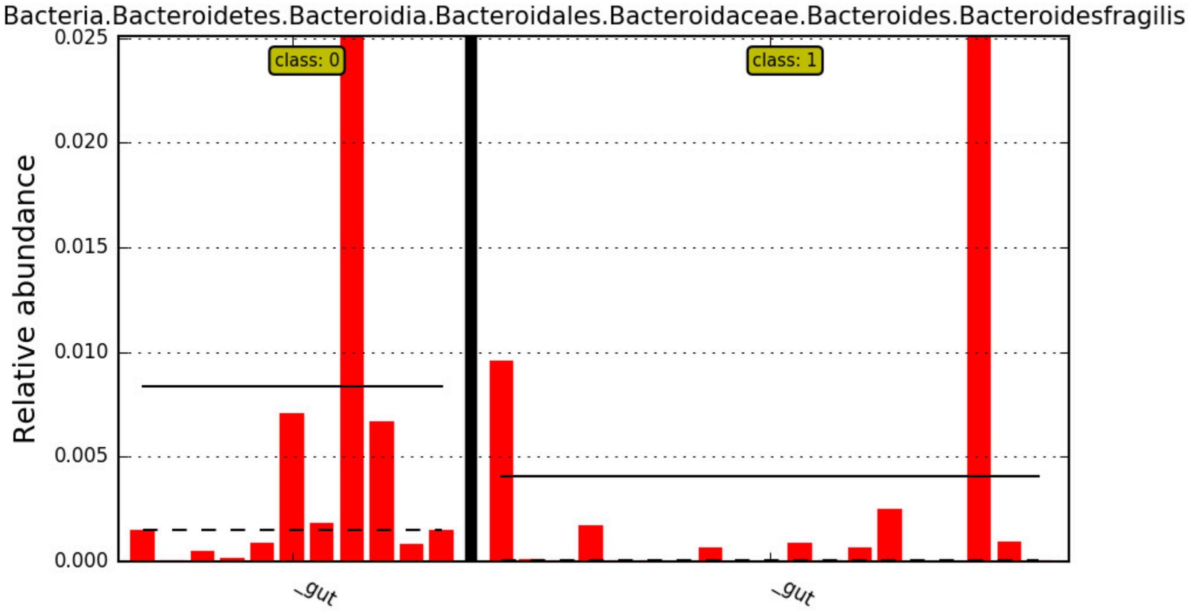
Bacteria.Bacteroidetes.Bacteroidia.Bacteroidales.Bacteroidaceae.Bacteroides.Bacteroidesfragilis
Relative abundance
0.025
0.020
0.015
0.010
0.005
0.000
class: 0
class: 1
_gut
_gut

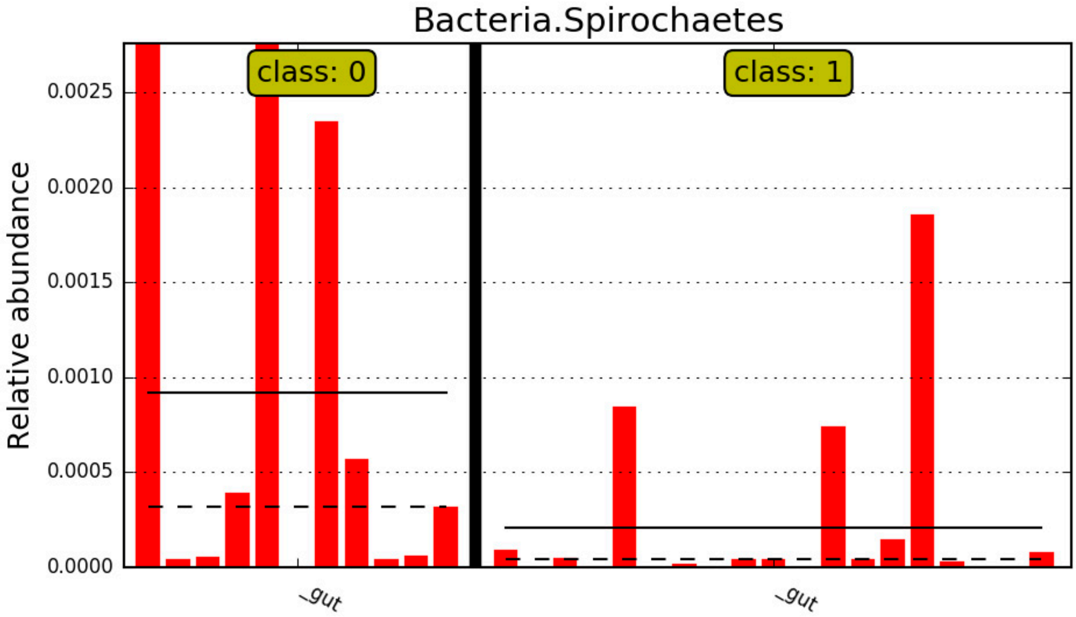

Figure S4. Differences in intestinal microbiota between Group 1 and Group 2 (1-Group 1; 0-Group 2).

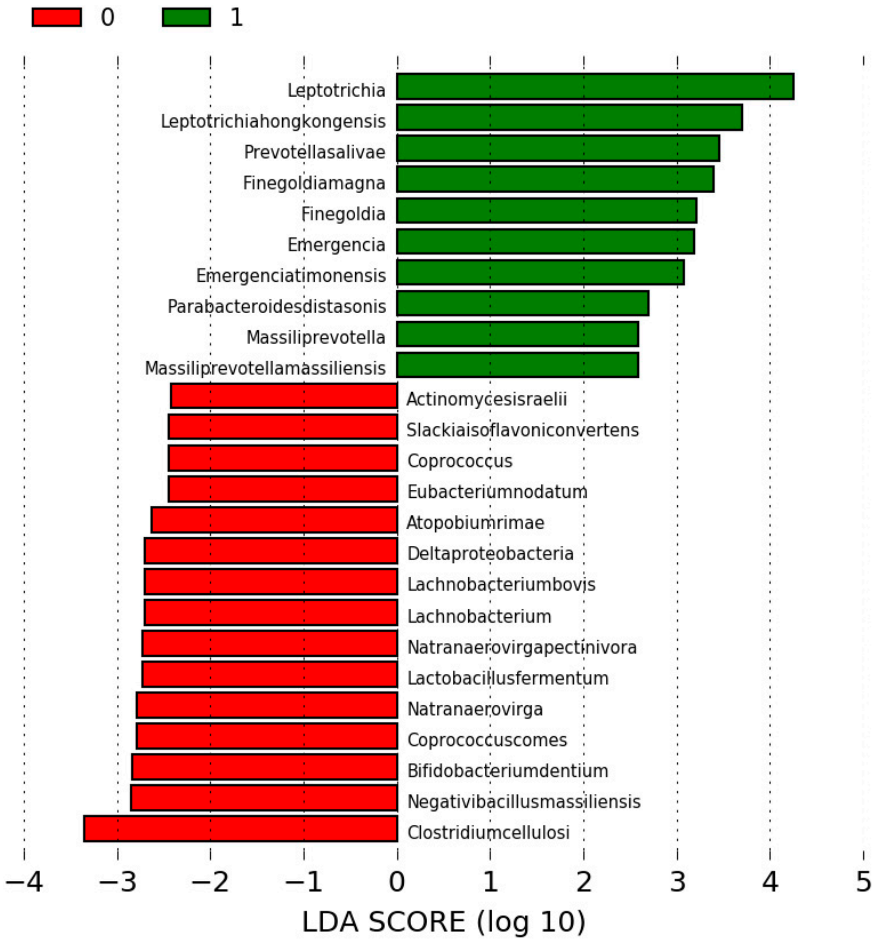
0
1
Leptotrichia
Leptotrichiahongkongensis
Prevotellasalivae
Finegoldiamagna
Finegoldia
Emergencia
Emergenciatimonensis
Parabacteroidesdistasonis
Massiliprevotella
Massiliprevotellamassiliensis
Actinomycesisraelii
Slackiaisoflavoniconvertens
Coprococcus
Eubacteriumnodatum
Atopobiumrimae
Deltaproteobacteria
Lachnobacteriumbovis
Lachnobacterium
Natranaerovirgapectinivora
Lactobacillusfermentum
Natranaerovirga
Coprococcuscomes
Bifidobacteriumdentium
Negativibacillusmassiliensis
Clostridiumcellulosi
−4 −3 −2 −1 0 1 2 3 4 5
LDA SCORE (log 10)

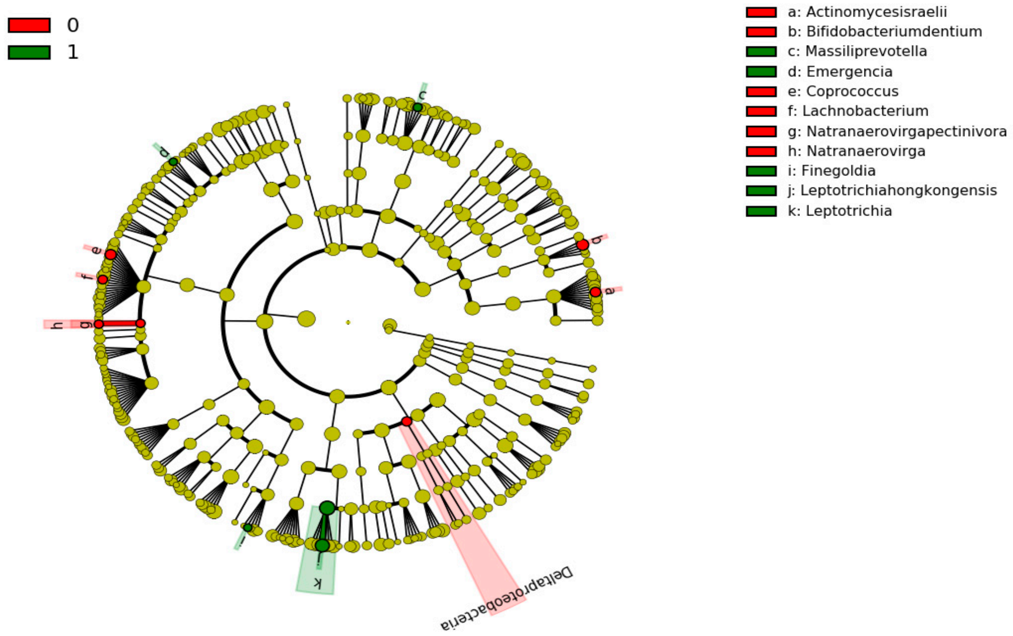
0
1
a: Actinomycesisraelii
b: Bifidobacteriumdentium
c: Massiliprevotella
d: Emergencia
e: Coprococcus
f: Lachnobacterium
g: Natranaerovirgapectinivora
h: Natranaerovirga
i: Finegoldia
j: Leptotrichiahongkongensis
k: Leptotrichia
Deltaproteobacteria

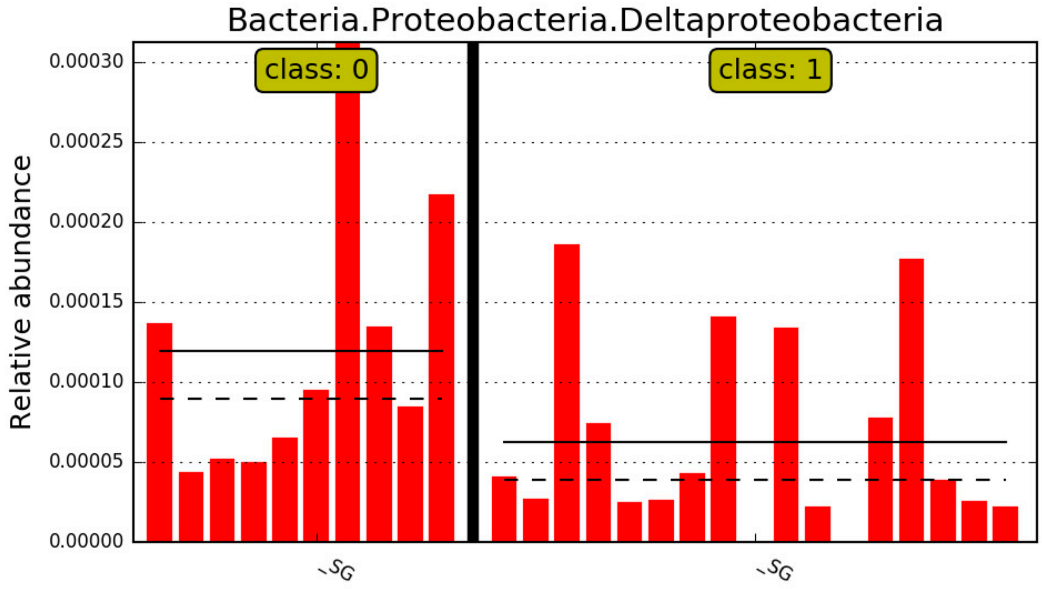

Figure S5. Differences in oral microbiota between Group 1 and Group 2 among patients undergoing SG (1-Group 1; 0-Group 2).

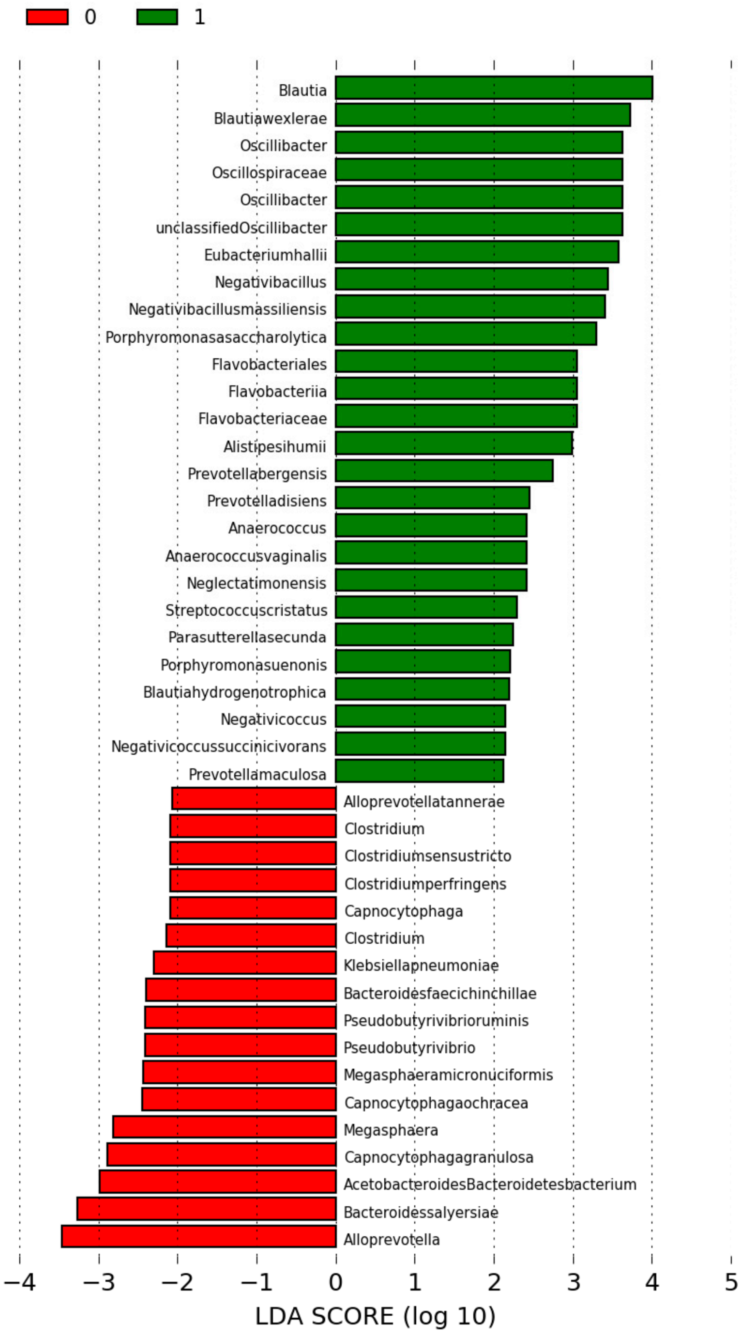

0
1
Blautia
Blautiawexlerae
Oscillibacter
Oscillospiraceae
Oscillibacter
unclassifiedOscillibacter
Eubacteriumhallii
Negativibacillus
Negativibacillusmassiliensis
Porphyromonasasaccharolytica
Flavobacteriales
Flavobacteriia
Flavobacteriaceae
Alistipesihumii
Prevotellabergensis
Prevotelladisiens
Anaerococcus
Anaerococcusvaginalis
Neglectatimonensis
Streptococcuscristatus
Parasutterellasecunda
Porphyromonasuenonis
Blautiahydrogenotrophica
Negativicoccus
Negativicoccussuccinicivorans
Prevotellamaculosa
Alloprevotellatannerae
Clostridium
Clostridiumsensustricto
Clostridiumperfringens
Capnocytophaga
Clostridium
Klebsiellapneumoniae
Bacteroidesfaecichinchillae
Pseudobutyrivibrioruminis
Pseudobutyrivibrio
Megasphaeramicronuciformis
Capnocytophagaochracea
Megasphaera
Capnocytophagagranulosa
AcetobacteroidesBacteroidetesbacterium
Bacteroidessalyersiae
Alloprevotella
−4
−3
−2
−1
0
1
2
3
4
5
LDA SCORE (log 10)

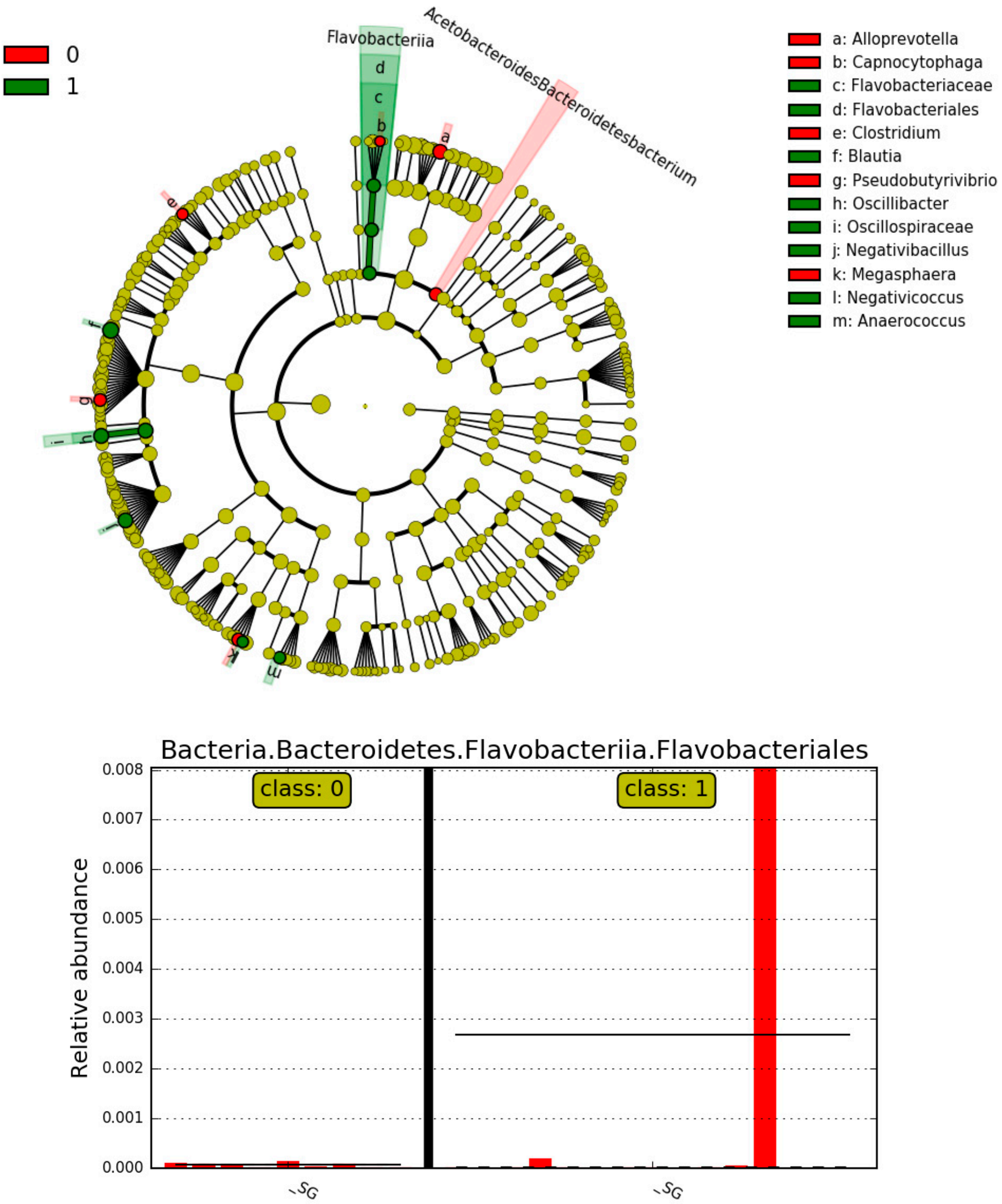

Figure S6. Differences in intestinal microbiota between Group 1 and Group 2 among patients undergoing SG (1-Group 1; 0-Group 2).

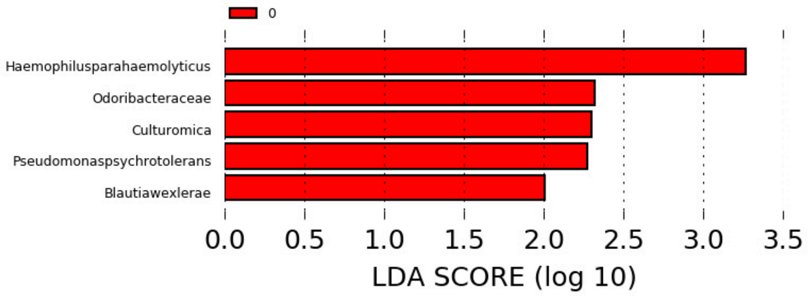

0
Haemophilusparahaemolyticus
Odoribacteraceae
Culturomica
Pseudomonaspsychrotolerans
Blautiawexlerae
0.0
0.5
1.0
1.5
2.0
2.5
3.0
3.5
LDA SCORE (log 10)

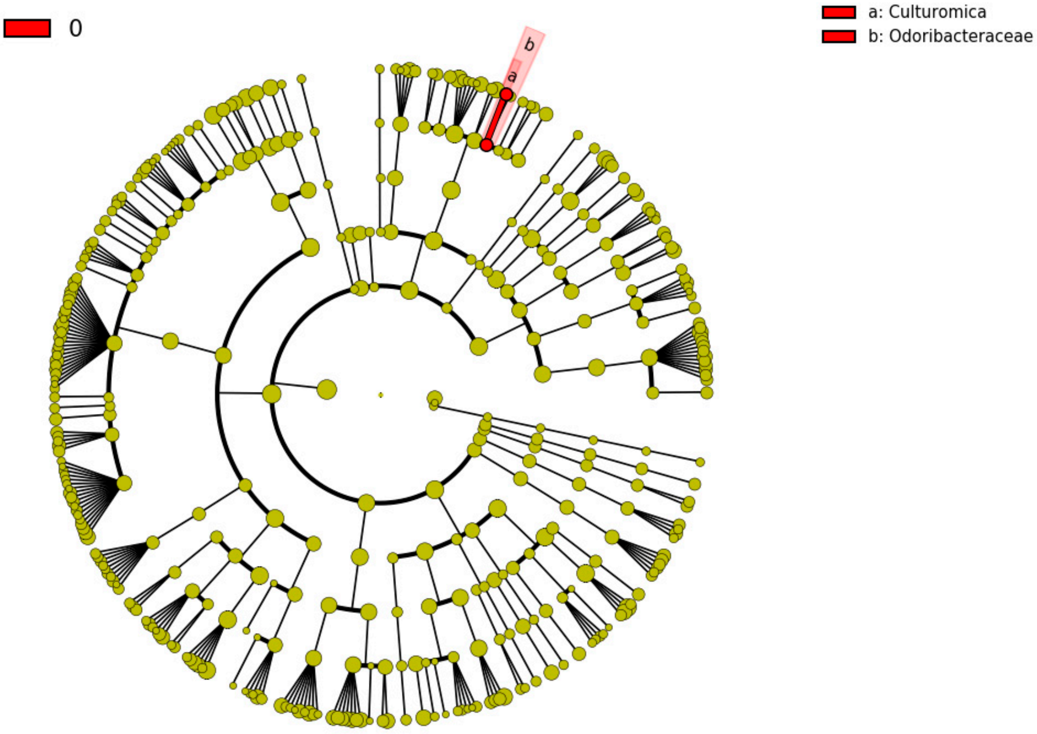

0
a: Culturomica
b: Odoribacteraceae
a
b

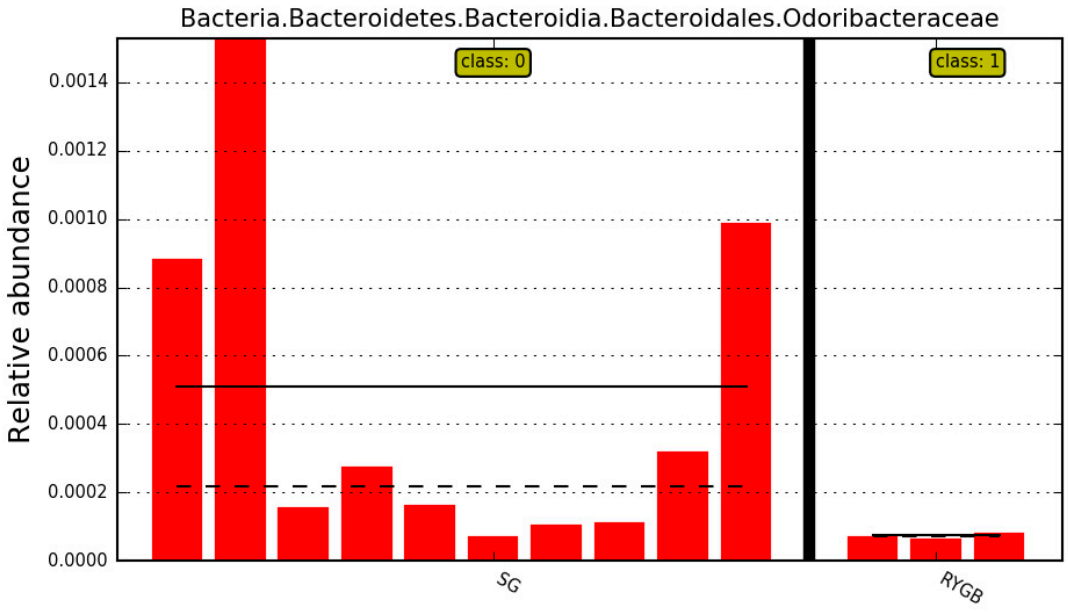

Figure S7. Differences in oral microbiota between Group 1 and Group 2 among patients undergoing RYGB (0-Group 1).

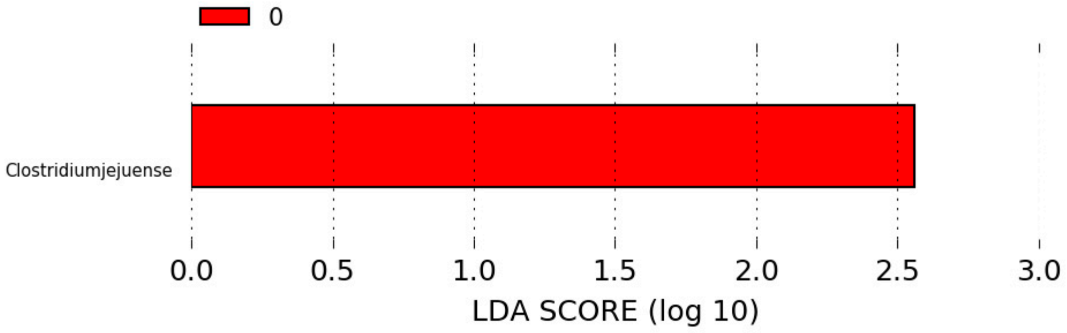

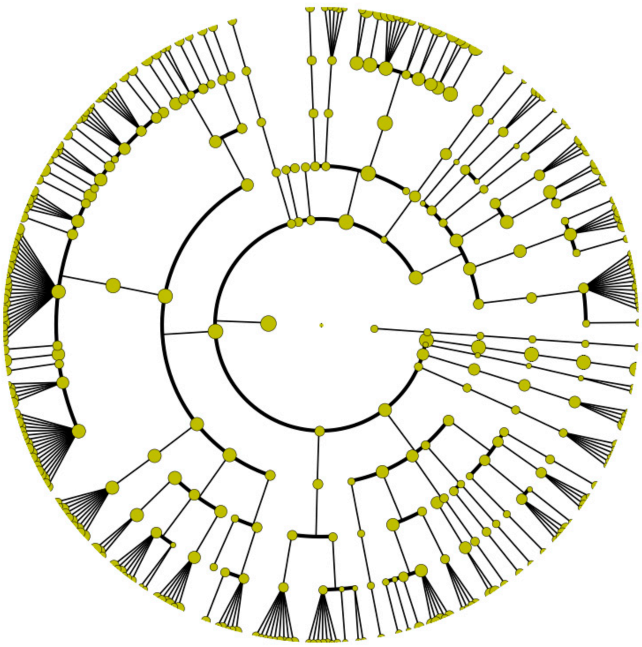

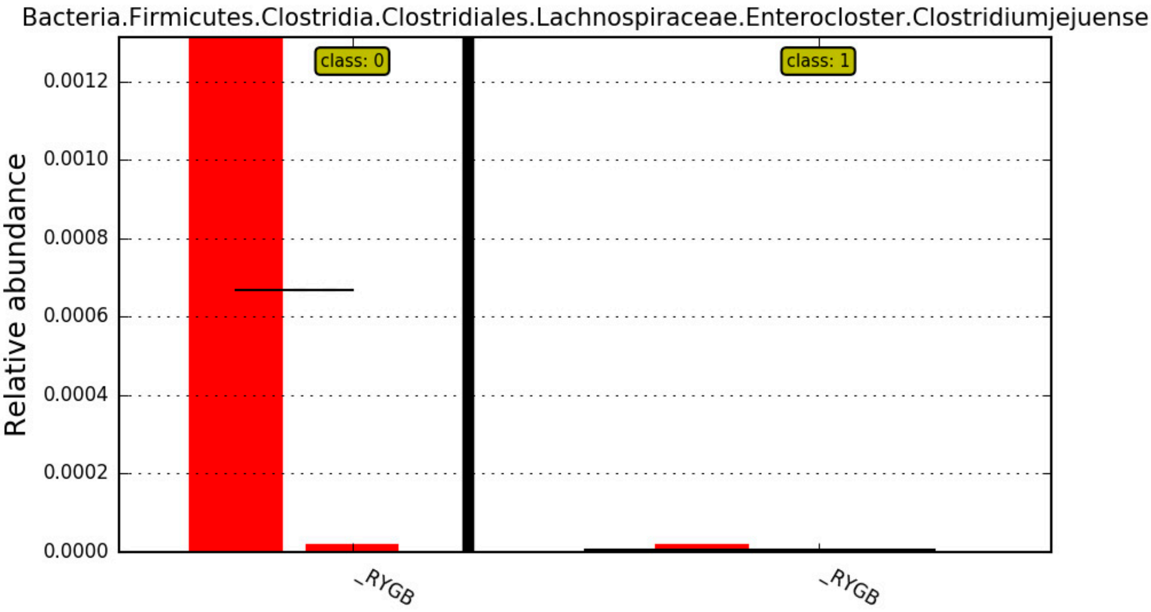

Figure S8. Differences in intestinal microbiota between Group 1 and Group 2 among patients undergoing RYGB (0-Group 1).
